# Use of Functional Near Infrared Spectroscopy to Assess Syntactic Processing by Monolingual and Bilingual Adults and Children

**DOI:** 10.3389/fnhum.2021.621025

**Published:** 2021-02-03

**Authors:** Guoqin Ding, Kathleen A. J. Mohr, Carla I. Orellana, Allison S. Hancock, Stephanie Juth, Rebekah Wada, Ronald B. Gillam

**Affiliations:** ^1^School of Teacher Education and Leadership, Utah State University, Logan, UT, United States; ^2^Department of Communicative Disorders and Deaf Education, Utah State University, Logan, UT, United States; ^3^Department of Psychology, Utah State University, Logan, UT, United States; ^4^Department of Speech-Language Pathology, Francis Marion University, Florence, SC, United States

**Keywords:** syntactic processing, bilingualism, semantic implausibility, language development, fNIRS neuroimaging, canonical sentences, relative clause, Chinese-English bilinguals

## Abstract

This exploratory study assessed the use of functional Near Infrared Spectroscopy (fNIRS) to examine hemodynamic response patterns during sentence processing. Four groups of participants: monolingual English children, bilingual Chinese-English children, bilingual Chinese-English adults and monolingual English adults were given an agent selection syntactic processing task. Bilingual child participants were classified as simultaneous or sequential bilinguals to examine the impact of first language, age of second-language acquisition (AoL2A), and the length of second language experience on behavioral performance and cortical activation. Participants were asked to select the agent of four types of sentences: subject-verb-object (SVO), passive (PAS), subject-extracted relative clause (SR), and object-extracted relative clause (OR) adopted from the “Whatdunit” task by Montgomery et al. (2016). Semantic cues were removed by using inanimate nouns for agents and patients, which constrained participants to make decisions based on syntactic knowledge. Behavioral results showed greater accuracy for canonical SVO and SR sentence types than for noncanonical OR and PAS sentence types, which aligns with prior studies. Neuroimaging results revealed greater hemodynamic responses to relative clauses (i.e., SR and OR sentences) than to simple sentences (SVO and PAS), especially for Chinese-English bilinguals suggesting first-language transfer influencing sentence processing in English. The effects AoL2A and the length of second language experience showed no significant differences between simultaneous and sequential bilinguals or between bilingual adults and children for identifying the correct agent in each sentence. However, neuroimaging results demonstrated greater hemodynamic responses in right dorsolateral prefrontal cortex (DLPFC) and left inferior parietal lobule (IPL) in simultaneous bilinguals compared to sequential bilinguals and greater hemodynamic responses in left and right DLPFC and left IPL among bilingual adults. Different behavioral and neural hemodynamic response patterns afford new insights into the effects of syntactic knowledge on sentence processing.

## Introduction

Bilingualism describes the regular use of more than one language, which has become a worldwide phenomenon (Grosjean, 2008). The appeal of bilingualism and its possible benefits have fostered increased attention from a variety of audiences. Over the past two decades, research using newer neuroimaging methods to study language processes has increased dramatically and the question of how bilinguals process a second language (L2) has become a central issue (Yokoyama et al., 2006; Buchweitz et al., 2012). It has been proposed that bilinguals process an L2 through the same mechanism underlying first-language (L1) processing (Abutalebi, 2008; Waldron and Hernandez, 2013). However, it remains largely unknown as to how bilinguals process an L2 that differs markedly from their L1. For example, Korean, Arabic, and Chinese, which are outside the Indo-European language family, are much more different compared to English than languages that are in the family, such as Dutch and Spanish (Walqui, 2000). Languages are commonly compared in terms of phonological, morphological, and semantic characteristics, but differences in syntax and pragmatics also merit attention. Neuroscience research methods can target such syntactical variations to better understand how bilingual brains process different languages at the sentence level.

Researchers have used various methodologies such as functional magnetic resonance imaging (fMRI) (e.g., Caplan, 2001; Yokoyama et al., 2006), electroencephalograph (EEG) (e.g., Weber-Fox and Neville, 1996; Garcia et al., 2018), magnetoencephalography (MEG) (e.g., Blanco-Elorrieta and Pylkkanen, 2015; Pellikka et al., 2015), and functional near-infrared spectroscopy (fNIRS) (e.g., Scherer et al., 2012; Jasińska and Petitto, 2013) to investigate L2 processing of bilinguals. In the past few decades, the use of fNIRS technology to investigate brain functions in neuroimaging research has increased rapidly (Pinti et al., 2018). fNIRS is safe, non-invasive and relatively inexpensive. Compared to other neuroimaging technologies such as fMRI, EEG and MEG, fNIRS has several advantages such as being highly portable, less susceptible to bodily movements, and suitable for a wide range of populations (Scherer et al., 2012; Pinti et al., 2018). Research has shown that fNIRS studies targeting syntactical processing have corroborated previously reported neuroimaging results using other technologies, such as fMRI and MEG (Kovelman et al., 2008b; Scherer et al., 2012; Jasińska and Petitto, 2013). However, little is known about how bilinguals process L2 sentences that are structurally different from those in their L1. For instance, English relative clauses (RCs) follow the head nouns they modify (e.g., The person who was nice to me likes to ride his mountain bike.), whereas Chinese RCs are head-final structures in which RCs appear before the head nouns (Huang and Liao, 2007) (shown in Example 1 and 2 below).

Prior studies targeting sentence processing have mainly included tasks requiring sentence listening or sentence reading. Research has shown that neural information in auditory sentence processing is transmitted from the primary auditory cortex (i.e., superior temporal gyri; STG) of the two hemispheres to the ventrolateral prefrontal cortex (Stankova et al., 2020). The transmitted pathways integrate areas including STG, parietal associated cortex, middle temporal gyrus (MTG), and prefrontal cortex (Friederici, 2015; Nasios et al., 2019). Activation has been most observed in regions in the left hemisphere, such as left inferior frontal gyrus (IFG), STG, MTG, and inferior parietal lobule (IPL) (Vigneau et al., 2006).

In the sentence processing literature, Broca's area and Wernicke's area are associated with speech production, perception, and comprehension (Rizzolatti and Arbib, 1998). Additionally, research has suggested that during sentence processing STG may play a role in “comparing the incoming information with information stored in long-term memory” (Stankova et al., 2020, p. 334). During sentence comprehension, left IPL is proposed to be associated with verbal working memory (Meyer et al., 2012). Prior studies have shown that cortical regions including STG and IPL are late-maturing structures that continue to develop (between 9 and 15 years of age) (Lenroot and Giedd, 2006; Shaw et al., 2008). In addition to language related areas, cortical regions associated with cognitive control such as dorsolateral prefrontal cortex (DLPFC) and medial prefrontal cortex (MPFC) are also involved for sentence processing (Shaw et al., 2008; Stankova et al., 2020). According to Krawczyk (2002), the DLPFC is associated with executive function and involved in higher order processes including manipulating relevant information, making conscious decisions, maintaining decision goals, considering options, and predicting outcomes. Similarly, the MPFC also plays a role in decision making such as monitoring conflicts, detecting errors, and executive control (Euston et al., 2012). Therefore, examining neural changes of cortical regions including left IFG, left STG, left IPL, DLPFC, and MPFC is critical in understanding sentence processing.

In studies targeting syntax and syntactical variations, RC structures have been widely used to examine sentence processing in monolinguals and bilinguals (Caplan, 2001, 2007; Kovelman et al., 2008a; Jasińska and Petitto, 2013). The two common types of RCs most often compared are subject-extracted relative clauses (SRs, as shown in Example 1 below) and object-extracted relative clauses (ORs, as shown in Example 2 below) (Keenan and Comrie, 1977; Gibson and Wu, 2013). Because SRs in English follow the canonical thematic templates with the unmarked agent-verb-patient order, processing SRs is considered less effortful than processing ORs for English speakers, which are in the noncanonical patient-agent-verb order (Kovelman et al., 2008a; Jasińska and Petitto, 2013). Kovelman et al. (2008a) and Jasińska and Petitto (2013) found that both monolinguals and bilinguals showed greater neural recruitment for ORs as compared with SRs. However, in Chinese, ORs follow the dominate agent-verb-patient order, as shown in the following examples:

1. SR: 帮助学生的张老师今天没来。
  Help the student **Ms. Zhang** today not come. (literal translation)
  **Ms. Zhang** who helps the student didn't come today. (interpretation)
2. OR: 张老师帮助的学生今天没来。
  Ms. Zhang help **the student** today not come. (literal translation)
  **The student** whom Ms. Zhang helps didn't come today. (interpretation)

In the Chinese examples above, RCs are placed initially and the SR is in verb-patient-agent order instead of following the canonical thematic templates of English. Moreover, as illustrated, RCs in Chinese tend to be shorter and less complex compared with English (Lin, 2011). Lin (2011) conducted a textual analysis to compare relative clauses in Chinese or English original texts and their corresponding English or Chinese translations. The author found that on average Chinese texts (i.e., originals or the Chinese translations from English) contained 6.63 or 7.32 syllables, respectively, as compared to 11.28 or 13.47 syllables in English texts (originals or English translation from Chinese). The average number of relative clauses embedded in other relative clauses in English texts was four or three whereas the average number of embedded clauses in Chinese texts was zero. It is recognized that such differences, including the structure of the L1 may influence sentence processing in the L2. Because of the differences between English and Chinese RCs in placement, length, and word-order, it is reasonable to assume that Chinese bilinguals may evidence some difficulty when processing SRs in English. Indeed, these bilinguals may rely on their L1 structure and have more difficulty processing SRs than ORs.

In addition to SRs and ORs, subject-verb-object (SVO) and passive (PAS) structures are two other commonly compared sentence types in studies of sentence processing among monolinguals and bilinguals (Yokoyama et al., 2006). Because SVO sentences that follow the agent-verb-patient order are considered as canonical sentence types, processing SVO sentences is supposedly easier than processing PAS sentences, which follow the noncanonical patient-verb-agent order. Similar to English, SVO sentences in Chinese follow agent-verb-patient order, however, PAS sentences in Chinese follow patient-agent-verb order and the passive construction and tense inflections of the verb are lacking (shown below).

3. 猫被狗追。
  Cat **by** dog chase. (literal translation)
  The cat was chased by the dog. (interpretation)

In the Chinese example above, instead of using the passive construction (i.e., be + past participle), Chinese PAS sentences use a marker (i.e., 被) to indicate a passive voice. Because the passive marker highlights the agent as the noun followed by the marker, identifying the agent in Chinese PAS may not require more effort than that of Chinese SVO (Huang and Liao, 2007). If Chinese bilinguals rely on their L1 structures to process English, it remains undetermined as to how bilinguals process English PAS sentences, which include English grammatical features (e.g., the passive construction including “to be” and inflected verbs) not found in Chinese.

Using more than one language on a regular and developing basis could have a profound impact on both the organization and function of a human brain (Newman et al., 2001; Abutalebi et al., 2015; Grundy et al., 2017). The development of an L2 reportedly corresponds with greater gray matter volume at the cortical level, greater integrity of white matter, and stronger functional connectivity between brain regions (see review of Grundy et al., 2017). The degree of neural changes may be dependent on age of L2 acquisition (AoL2A) (Kim et al., 2004; Jasińska and Petitto, 2013; Berken et al., 2015) and longer vs. shorter L2 experience (Bialystok et al., 2012; Jasińska and Petitto, 2014). Studies of bilinguals with early vs. late L2 onset have examined the effects of AoL2A on the neural basis of language processing and have shown that early L2 exposure can have a profound impact on the developing brain. For example, more activation of the left IFG was found in late bilingual groups as compared to early bilingual groups with picture naming and sentence production tasks (Kim et al., 2004). Jasińska and Petitto (2013) found that time of exposure to two languages early also influenced neural systems associated with language areas (e.g., left IFG) and cognitive control regions (e.g., DLPFC). In their study, Jasińska and Petitto (2013) used a sentence judgement task with monolingual, early bilingual, and late bilingual children and adults. Results showed that late bilingual children evidenced more activation in areas such as left IFG, right IFG, left STG, and DLPFC as compared to early bilingual children.

Life-long L2 experience may also yield structural changes in language-associated brain regions. For example, by comparing younger bilinguals (ages 6–8) with older bilinguals (ages 8–10) in a single-word reading task, Jasińska and Petitto (2014) found that younger bilinguals evidenced stronger STG activation when reading both irregular and regular words. However, older bilinguals recruited stronger IFG and IPL when reading irregular words compared with regular words. The authors argue that older bilinguals who are more skilled in reading showed greater activation of neural areas associated with morphology, syntax and semantics, which is related to whole-word processing. Whereas, less skilled younger bilinguals showed greater activation of neural areas associated with phonological processing, which is related to matching sounds onto corresponding letters. It has been found that young bilinguals with shorter L2 language experience tended to recruit greater activation of frontal areas [e.g., anterior cingulate cortex (ACC) and DLPFC] compared to monolinguals, perhaps as a means to deal with language conflict (Abutalebi et al., 2012; Abutalebi and Green, 2016). Research has also shown that older bilinguals with longer L2 experience and higher proficiency processed L2 in ways similar to monolinguals (Yan et al., 2016). The question as to whether bilinguals with longer L2 exposure perform similarly or differently to young simultaneous/early bilinguals on varied language tasks has only been minimally explored. Including monolinguals as a control group and comparing both bilingual groups with monolinguals could illuminate comparisons of neural system changes associated with AoL2A and length of L2 experience, beyond the direct comparison between older bilingauls and young simultaneous bilinguals.

We sought to better understand the potential for L1 transfer when processing syntactical variation among users of two very different languages. We considered if and how neural activation patterns differ among Chinese-English bilinguals (simultaneous vs. sequential; children vs. adults) on a syntactic processing task. The study sought to specifically examine whether AoL2A influences behavioral performances and cortical activation patterns by comparing simultaneous and sequential bilinguals in a sentence-processing task. Another question targeted whether longer and shorter exposures to L2 correspond with changes in neural systems, so we compared simultaneous bilinguals, sequential bilinguals, and bilingual adults. Meanwhile, monolinguals were used as control groups to investigate effects of AoL2A and length of L2 experience on syntactical processing of sentence types.

## Materials and Methods

This present study recruited four groups of participants: Chinese-English bilingual chidren and adults, and English monolingual children and adults. The bilingual children were further divided into two groups: simultaneous (exposure to L2 from birth) and sequential (exposure to L2 after L1 acquisition) bilingual groups. We used four sentence types (SVO, PAS, SR, and OR) to investigate neural activation patterns among Chinese-English bilinguals on an auditory agent-selection syntactic task. All participants were asked to select the agent of a set of English sentences presenting the four sentence types. In line with previous language processing studies, bilinguals were predicted to perform less accurately and recruit greater neural activation for PAS and OR (noncanonical) sentence types relative to SVO and SR, which are canonical. However, if L1 Chinese transfer is a factor that influences sentence processing of English (L2), bilinguals were predicted to show more difficulty and greater neural activation in SR and OR relative to SVO and PAS which are similar to Chinese SVO and PAS structures.

### Participants

Fifteen 9–12 year-old monolingual English children (female = 11, mean age =11.2) and 16 Chinese-English bilingual children, ages 9–12 (female = 8, mean age = 11.2) participated in the study. Eight of the bilingual children were identified as simultaneous bilinguals (being exposed to two languages from birth; female = 5, mean age = 11.6) and the other eight bilinguals were identified as sequential bilinguals (being exposed to English between the ages of 3–7; female = 3, mean age = 10.7). Participants also included seven monolingual English adults (female = 4, mean age = 22.8) and 12 late Chinese-English bilingual adults (female = 4, mean age = 26.5; being exposed to English between ages of 6–11).

Parents of child participants and all adult participants were asked to complete a questionnaire indicating their socio-economic status (SES) as indexed by maternal education and family income, basic developmental and educational information including whether or not they use another language and age of second language acquisition, and any medical issues they might have. All participants were right-handed, healthy with normal or corrected-to-normal vision, normal hearing, with no known cognitive deficits or any speech/language disorders or reading disabilities. Maternal education was coded on a scale of 1–5 from “high school” to “professional degree (e.g., RN, Ph.D.).” Family annual household income was coded on a scale of 1-11, which were 1) 0-$7,000, 2) $8,000-$12,000, 3) $13,000-$15,000, 4) $16,000-$19,000, 5) $20,000-$22,000, 6) $23,000-$25,000, 7) $26,000-$29,000, 8) $30,000-$36,000, 9) $37,000-$50,000, 10) $51,000-$75,000, and 11) $76,000. No differences were found among child participants in age and family income, but a significant difference in maternal education was found [F_(2,29)_ = 14.43, *p* < 0.001]. Mean maternal education for monolingual children was 2.5, which was significantly lower than both simultaneous bilingual children (*M* = 4.1) and sequential bilingual children (*M* = 4.0). All bilingual participants indicated using more than 3 h of both languages every day. All participants or parents of participants signed IRB approved consent forms and received minimal monetary compensation.

### Behavioral Tests for Child Participants

All child participants were asked to complete a battery of English tests to assess their English proficiency, short-term symbolic memory, and auditory working memory. All adult participants were undergraduate or graduate students at the local university and were asked to complete an auditory working memory test. All bilingual adults had studied English for more than 10 years and met the local university's TOFEL requirements, thus confirming their proficiency sufficient to complete the tasks in the study.

#### Language Proficiency

English proficiency was measured with the Grammaticality Judgement (GJ) task of the Comprehensive Assessment of Spoken Language-2nd Ed. (CASL-2; Carrow-Woolfolk, 2017). During the GJ tasks, the administrator provided sentences orally without supplying any pictures and the participant was asked to identify grammatically incorrect or correct sentences. Following any ungrammatical sentence identification, the participant was asked to fix the sentence by changing one word without altering overall sentence meaning. Scores were calculated according to the scoring rules.

#### Short-Term Symbolic Memory

The Symbolic Memory (SyM) of the Universal Nonverbal Intelligence Test-2nd Ed. (UNIT2; Bracken and McCallum, 2015) was used to measure short-term memory. The examiner used the eight universal administration gestures and administered the task completely nonverbally. SyM includes 10 response cards (5 green, 5 black), which depicts a sequence of universal symbols for baby, girl, boy, woman, and man, arranged according to the participant's dominant hand. After 5 s of exposure to a sequence of the universal human symbols presented on a stimulus plate, the participant was required to recreate the sequence of the universal human symbols. A raw score of one point was given on correct response. The examiner discontinued the task after three consecutive incorrect scores. Scores were determined according to the scoring rules.

#### Working Memory

Working memory was tested by the Auditory Working Memory (AWM) subset of the Woodcock-Johnson Tests of Cognitive Abilities (WJ-III; Woodcock et al., 2005). The WJ-III is an assessment for participants ages 2–90. The examiner asked the participant to listen to trials with numbers and object names in a random order. The participant needed to repeat back each trial in the respective orders. The task begins with two items (single object and single number) and increases to eight items (4 objects and 4 numbers). The participant received one point for the correct sequence of objects and another one point for numbers. A possible raw score of two points is given on each trial. Final scores were determined according to the scoring rules.

### Stimuli

This study used an agent-selection task, known as “Whatdunit?” (Montgomery et al., 2016). In this paradigm, participants listen to sentences and point to the picture of the agent. The nouns in the sentences are inanimate objects, which constrains the participants to make decisions based on syntactic knowledge or linguistic cues. Participants responded to a total of 48 trials, including four sentence types: subject-verb-object (SVO), passive (PAS), subject relatives (SR), and object relatives (OR). Each sentence type was presented in 12 sentences controlling for sentence length and vocabulary complexity (e.g., SVO: *The ring moved the square behind the very bright cold bed*; SR: *The fork that wiped the boot near the shirt was bright*; OR: *The hat that the car fixed under the fork was hot;* PAS: *The ring was bathed by the key under the hot bread*).

Among the four sentence types, SVO and SR are canonical sentence types in which the agent (the noun that is doing the action) appears first. The OR and PAS sentences are non-canonical sentence types, in which the patient other than the agent appears first. The SVO and PAS items are simple sentences with a single clause, whereas the OR and SR are complex sentences with two clauses. Sentences were presented in two blocks with 24 items (6 SVO, 6 SR, 6 OR, and 6 PAS) in each block in a pseudo-random order.

### Procedures

**Figure 1:**
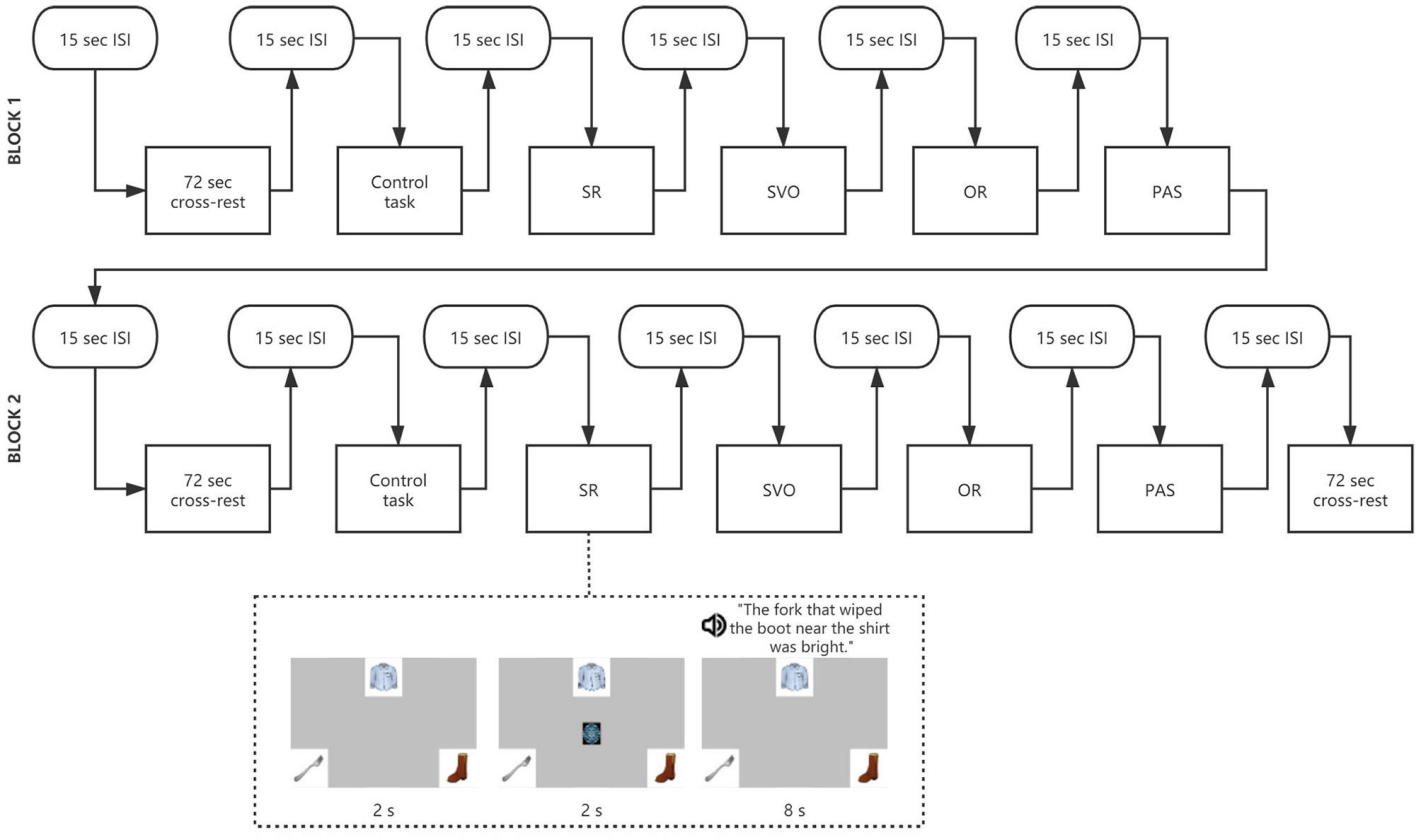
The experimental design and examples of stimuli used in the experimental task.

Participants completed all tasks individually during lab visits. During the experimental tasks, participants sat ~55–60 cm from a computer monitor and listened to each sentence with three pictures shown on the screen. They were instructed to select the agent of the sentence by clicking the picture of the agent as quickly as possible after listening to the whole sentence. Three pictures corresponding to the three nouns in each sentence trial were displayed on random sides of the screen. At the start of each item, a colorful picture appeared in the center of the screen for 2 s to draw participants attention to the middle of the screen. After 2 s, the colorful design disappeared and the sentence trial was presented auditorily with the three pictures presented (see Figure 1). Each block was separated by a 72 s rest period and a 15 s ISI (inter-stimulus interval) was presented prior to the onset of each sentence type. The whole onset time for each sentence was 8 s. The testing session for each participant was approximately 25 min including the NIRS signal quality checking and the practice of training items.

**Figure 2:**
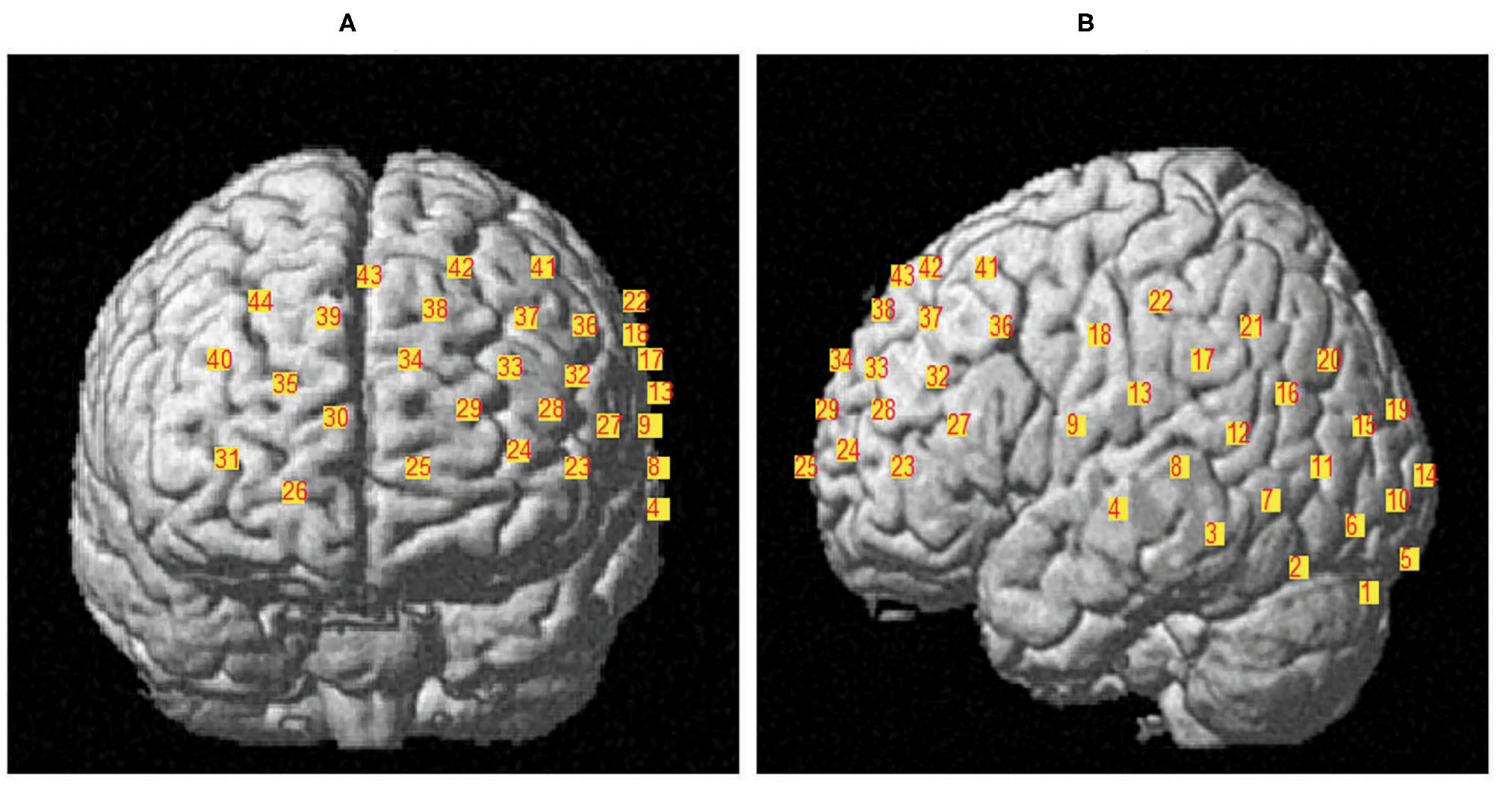
Example of one participant's fNIRS two 3 × 5 channel placement. Picture **(A)** shows the coverage of regions in frontal area and picture **(B)** shows the coverage of regions in left hemisphere. The mean channel location is delineated in Table 1.

This study used fNIRS to examine neural mechanisms underlying L2 processing of bilinguals. All participants completed two training items for each sentence type and then fNIRS scanning began. We used the Hitachi ETG-4000 (Hitachi Medical Co. Japan) with 44 channels divided across two 3 × 5 probe caps, acquiring data at 10 Hz. Two arrays were placed on the front and left sides of the participant's head and covered mostly the frontal area and the left hemisphere which included regions of interest (ROIs) pertinent to the study: left DLPFC, right DLPFC, MPFC, left inferior frontal cortex (IFC), left STG, and left IPL (See Figure 2). The signal quality was tested for under-gained or over-gained channels by using the NIR gain quality check before recording based on the Hitachi guidelines. Data were recorded at 695 and 830 nm. After the experiment, spatial coordinates were obtained for each participant using the Polhemus PATRIOT digitizing software following procedures consistent with previous studies (Wan et al., 2018; Ong et al., 2020).

**Table 1:** Mean channel locations for the fNIRS cap.

**Channels**	**MINI coordinates (x, y, z)**	**Brodmann area**	**Region of interest**	**Overlap**
CH08	80, 1, 54	22-Superior Temporal Gyrus	Left STG	0.69753
CH12	77, 16, 63	22-Superior Temporal Gyrus	Left STG	0.57325
CH16	72, 30, 75	39-Angular gyrus- part of Wernicke's area	Left IPL	0.625
CH17	77, 4, 79	40-Supramarginal gyrus part of Wernicke's area	Left IPL	0.57098
CH20	64, 45, 87	39-Angular gyrus- part of Wernicke's area	Left IPL	0.90323
CH21	73, 19, 91	40-Supramarginal gyrus part of Wernicke's area	Left IPL	0.56338
CH23	60, −77, 46	45-pars triangularis Broca's area	Left IFC	0.52688
CH24	39, −95, 46	10-Frontopolar area	MPFC	0.80377
CH25	10, −101, 40	10-Frontopolar area	MPFC	0.93069
CH26	−18, −98, 33	10-Frontopolar area	MPFC	0.56013
CH27	67, −61, 55	45-pars triangularis Broca's area	Left IFC	0.9375
CH28	51, −85, 60	46-Dorsolateral prefrontal cortex	Left DLPFC	0.7098
CH29	23, −97, 56	10-Frontopolar area	MPFC	0.90602
CH30	−7, −99, 51	10-Frontopolar area	MPFC	1
CH31	−33, −91, 44	10-Frontopolar area	MPFC	0.87986
CH32	57, −69, 70	45-pars triangularis Broca's area	Left IFC	0.96629
CH33	35, −87, 70	46-Dorsolateral prefrontal cortex	Left DLPFC	0.96476
CH34	6, −95, 68	10-Frontopolar area	MPFC	0.66788
CH35	−21, −92, 61	10-Frontopolar area	MPFC	0.82657
CH36	61, −54, 83	44-pars opercularis- part of Broca's area	Left IFC	0.75627
CH37	42, −75, 84	9-Dorsolateral prefrontal cortex	Left DLPFC	0.6484
CH38	17, −88, 82	9-Dorsolateral prefrontal cortex	Left DLPFC	1
CH39	−12, −90, 77	9-Dorsolateral prefrontal cortex	Right DLPFC	0.94262
CH40	−36, −81, 67	46-Dorsolateral prefrontal cortex	Right DLPFC	0.89302
CH41	45, −61, 97	9-Dorsolateral prefrontal cortex	Left DLPFC	0.88608
CH42	24, −76, 96	9-Dorsolateral prefrontal cortex	Left DLPFC	0.5084
CH43	−0.8, −83, 92	9-Dorsolateral prefrontal cortex	Left DLPFC	0.59375
CH44	−27, −79, 83	9-Dorsolateral prefrontal cortex	Right DLPFC	1

### fNIRS Data Processing

Following Brigadoi et al. (2014), Fu et al. (2016), and Plichta et al. (2007), data were filtered according to wavelet-minimum description length (MDL, Gaussian low-pass FWHM at 4 s) and were precolored and prewhitened using Statistical Parametric Mapping for NIRS (NIRS-SPM) (Ye et al., 2009) to remove task-irrelevant noise and improve the signal-to-noise ratio. Then NIRS-SPM yielded concentration values for oxygenated hemoglobin (HbO), deoxygenated hemoglobin (HbR), and total hemoglobin (HbT), respectively, for each participant according to the Beer-Lambert equation. The 15 s ISI prior to the onset of each sentence type was used as a baseline correction to remove signal drift over time. Similar to Wan et al. (2018), we determined the starting and ending time points of the hemodynamic response functions individually for each ROI by fitting the signal according to a Fourier series with harmonics. NIRS-SPM was used to register the spatial coordinates of the channels to obtain Brodmann areas that provided the percentage of channel overlapping within a brain region. All channels with 50% or greater overlapping area within a region were averaged together to select channels for each ROI (Rorden and Brett, 2000; Wan et al., 2018), as delineated in Table 1, followed by taking the square root of the signal power of the entire channel to normalize the signal (Ong et al., 2020). Finally, area under the curve (AUC) was computed by using the standard trapezoid function in Matlab (Wan et al., 2018). AUC was selected as the dependent variable because it is more consistent with the nonlinear nature of the data and it represents the curve shape to better capture relative differences in hemodynamic response functions (see deoxy- and oxy-hemoglobin curves in Supplementary Data).

## Results

### Behavioral Tests Results for Children

Screening tests were used to assess English proficiency, short-term symbolic memory, and auditory working memory of child participants. A series of one-way ANOVAs were used to analyze the screening tests data with each test score [i.e., grammatic judgement (GJ), symbolic memory (SyM), or auditory working memory (AWM)] as dependent variables and language group (i.e., monolingual, simultaneous bilingual, and sequential bilingual) as an independent variable. For GJ, the analyses revealed a main effect of language group [monolingual vs. bilingual, *F*_(2,29)_ = 6.56, *p* = 0.016] and a main effect of AoL2A [simultaneous vs. sequential bilingual, *F*_(2,29)_ = 14.43, *p* < 0.001]. Both monolingual and simultaneous bilingual groups scored significantly higher than the sequential bilingual group on identifying grammatically incorrect or correct sentences. No significant differences were found for SyM and AWM (See Table 2).

**Table 2:** SES and screening tests score means for child participants.

**Group**	**Family income**	**Maternal education**	**GJ**	**AWM**	**SyM**
Monolingual (*n* = 15)	8.4	2.5^*^	55.2	23	16.5
Simultaneous Bilingual (*n* = 8)	9.6	4	54.1	25	16.1
Sequential Bilingual (*n* = 8)	7.8	4.1	29.3^*^	22	14.1

* *p <0.05*.

### Behavioral Results of the Sentence Processing Task

A series of repeated-measure ANOVAs were used to analyze behavioral data of the processing task with sentence types (i.e., SVO, SR, OR, and PAS) as a within-subject factor and group (i.e., monolingual children vs. bilingual children; monolingual adults vs. bilingual adults; or bilingual adults vs. bilingual children) as a between-subject factor. The dependent variable in these analyses was the accuracy (total number correct) on selecting the agent of each sentence.

For the comparison between monolingual and bilingual children, analyses revealed a main effect of sentence type [*F*_(1.64,42.69)_ = 12.55, *p* < 0.001]. *Post hoc* comparisons showed that the accuracy of canonical sentences (SVO and SR) was significantly higher than noncanonical sentence types (OR and PAS) for all groups. No main effects of language group, AoL2A, or interactions were found.

Comparison of the accuracy between monolingual and bilingual adults revealed significant sentence type x language group interaction [*F*_(1.69,21.99)_ = 3.8, *p* = 0.044). The accuracy for SVO, SR, and PAS was significantly higher than for OR for the bilingual group (*p* < 0.001). For the monolingual group, the accuracy for SVO was significantly higher than for PAS (*p* = 0.04). No main effect of sentence type or language group was found.

When we compared bilingual adults to bilingual children, we found a main effect of sentence type [*F*_(1.99,43.76)_ = 3.54, *p* = 0.037]. Accuracy on selecting the agent for SVO, SR, and PAS constructions was significantly higher than for OR, and the accuracy of SVO was significantly higher than PAS. No main effect of age group, or the interaction was found.

Overall, all participants performed more accurately for SVO and SR sentence types than for OR and PAS sentences. The accuracy of canonical sentence types was significantly higher than noncanonical sentences, which aligns with the findings of previous studies (see Table 3).

**Table 3:** Accuracy means for sentence types by language groups.

**Group**	**SVO**	**SR**	**OR**	**PAS**
Monolingual children	11.53 (0.83)	11.40 (0.91)	8.60(3.18)	9.53(2.39)
Simultaneous bilingual children	11.57 (0.54)	11.57 (0.79)	9.86(1.47)	11.14(1.07)
Sequential bilingual children	10.50 (2.00)	10.63 (2.13)	7.75(3.62)	7.50(3.99)
Monolingual adults	11.40 (0.89)	11.60 (0.55)	11.60(0.55)	11.80(0.45)
Bilingual adults	11.30 (0.82)	10.60 (1.17)	7.40(3.70)	10.80(1.69)

*Each sentence type includes 12 sentences and the participant receives one point for the correct identification of agent. A possible raw score of 12 points is given on each sentence type*.

### Neuroimaging Results of the Sentence Processing Task

The package lme4 in R (Bates et al., 2007) was used to build separate two-level nested generalized multilevel regression models (MLMs) for analyzing fNIRS data. The dependent variables were HbO AUC values for each brain region (i.e., left and right DLPFC and MPFC, left STG, left IPL and left IFC). Prior studies have shown that HbO changes have generally yielded more robust data and indicated spatially larger functional activation as compared with HbR values (e.g., Duong et al., 2000; Strangman et al., 2003). In this study, each sentence type constituted a unit within level one, associated with fixed effects for Sentence Type (i.e., SVO, SR, OR, and PAS), Sentence Form (canonical vs. noncanonical) and Sentence Structure (simple sentence vs. relative clause). These sentence types were nested within participants, who comprised the units of level two. By-participant random intercept with cross-level interaction MLMs were used.

The analysis of the power of the MLM models is complicated because conducting these analyses for a given sample size requires not only the effect size information, but also population values of all other parameters, including correlations and variance components (Kert and De Leeuw, 1998; Schoeneberger, 2016; Hox et al., 2018). Thus, different power values may be yielded in the study under same group comparisons because of varying group differences for each ROI activation (see Tables in Supplementary Data). Furthermore, as Hox et al. (2018) noted, most programs developed for MLM (e.g., PINT, Powerlmm, and SIMR) were designed for clustering randomized trials, which is not suitable for quasi-experimental designs. The current study used quasi-experimental design and sought to explore cross-level interactions. Thus, it is difficult to obtain plausible power values for each fit model in the study. However, separate power analyses were conducted for each fit model using the SIMR package, which is designed for analyzing power of MLM in R (Green and MacLeod, 2016).

#### Comparison Between Monolingual and Bilingual Children

**Figure 3:**
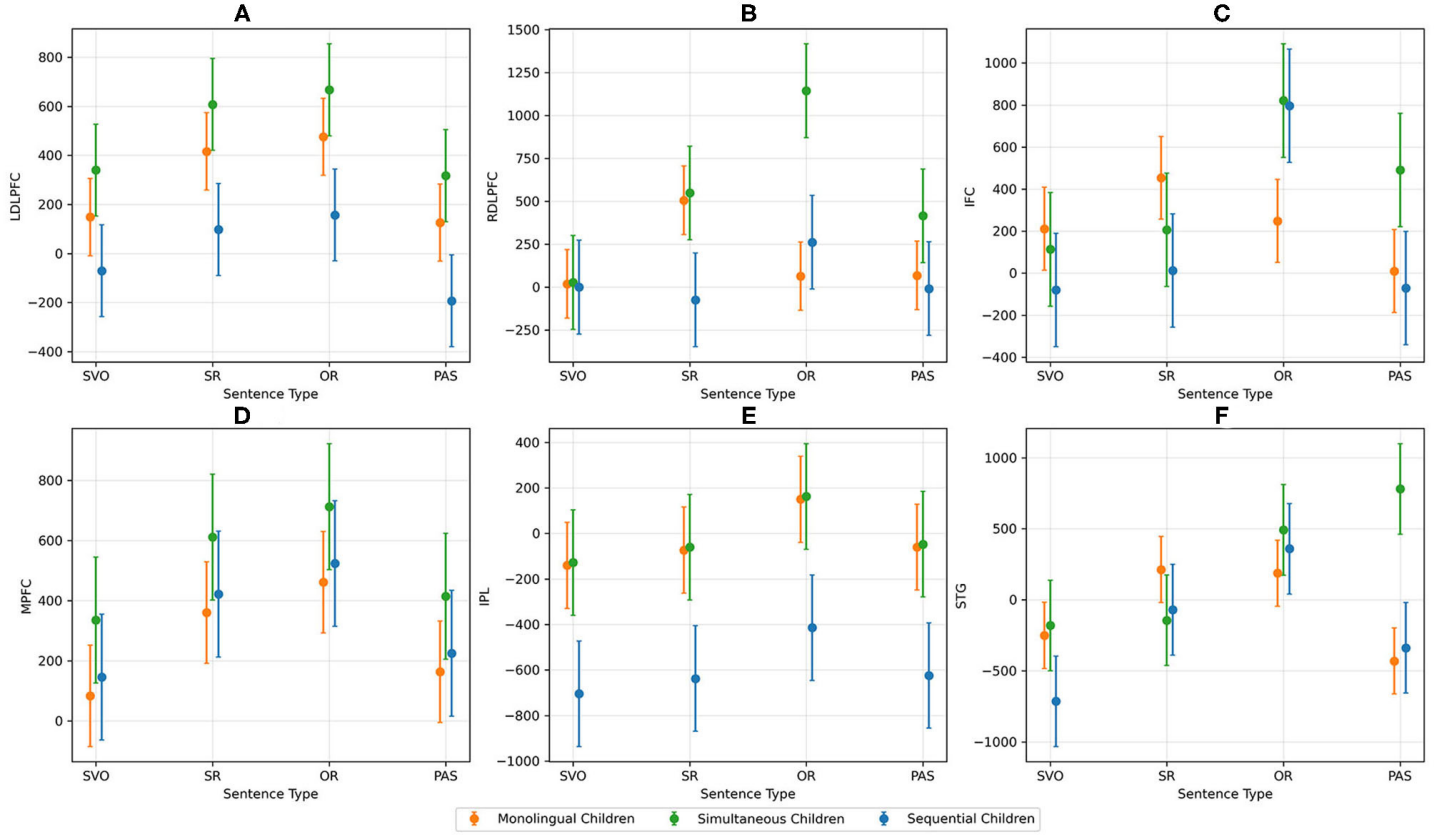
AUC activation for each ROI and sentence type across monolingual, simultaneous, and sequential children. Picture **(A)** shows the AUC activation for left DLPFC, **(B)** shows the AUC activation for right DLPFC, **(C)** shows the AUC activation for left IFC, **(D)** shows the AUC activation for MPFC, **(E)** shows the AUC activation for left IPL, and **(F)** shows the AUC activation for left STG.

Separate two-level MLMs were performed for child participants for each ROI exploring fixed factors including AoL2A, Language Group, Sentence Type, Sentence Form, Sentence Structure, and the interaction of Language Group x Sentence Type. Power ranged from 52.4 to 76.4% across the analyses. We found Sentence Structure was a significant predictor for left DLPFC [χ^2^(1) = 7.20; *p* < 0.01], right DLPFC [χ^2^(1) = 6.36; *p* = 0.012], MPFC [χ^2^(1) = 6.15; *p* = 0.013], left STG [χ^2^(1) = 7.69; *p* < 0.01], and left IFC [χ^2^(1) = 8.36; *p* < 0.01]. AoL2A was a significant predictor for left IPL [χ^2^(1) = 6.23; *p* = 0.013] and right DLPFC [χ^2^(1) = 5.49; *p* = 0.019). Sentence Type was a significant predictor for right DLPFC [χ^2^(1) = 4.04; *p* = 0.045], and Sentence Type x Language Group interaction was a significant predictor for right DLPFC [χ^2^(1) = 4.54; *p* = 0.033] and left IFC [χ^2^(1) = 5.22; *p* = 0.022], as delineated in Figure 3.

Overall, for Sentence Structure, greater activation was observed for RCs in almost all ROIs (left and right DLPFC, MPFC, left STG, and left IFC) compared with simple sentences, indicating that processing relatives clauses is more difficult than processing simple sentences. The significant interaction demonstrated greater activation of right DLPFC and left IFC in bilinguals for ORs compared with SVO and PAS sentence types. Additionally, compared to the monolingual children, bilingual children showed greater activation for OR in right DLPFC and left IFC. Simultaneous bilinguals evidenced greater activation in right DLPFC and left IPL than sequential bilingual children. Monolinguals demonstrated greater neural activation in left IPL for all sentence types compared to sequential bilingual children. Greater activation was observed for OR compared with SVO in right DLPFC (see Table 4).

**Table 4:** Main effects of sentence structure, AoL2A, sentence type and sentence type x language group interaction across ROIs for monolingual and bilingual children.

**ROI**	**Sentence structure**	**AoL2A**	**Sentence type**	**Sentence type x language group**
Left DLPFC	RCs > Simple Sentences (*p* < 0.01)			
Right DLPFC	RCs > Simple Sentences (*p* = 0.012)	Simultaneous > Sequential (*p* = 0.019)	OR > SVO (*p* = 0.045)	Bilingual > Monolingual in OR (*p* = 0.01); OR > SVO (*p* < 0.01) and PAS (*p* = 0.02) in bilingual
MPFC	RCs > Simple Sentences (*p* = 0.013)			
Left STG	RCs > Simple Sentences (*p* < 0.01)			
Left IPL		Simultaneous > Sequential (*p* = 0.013) Monolingual > Sequential (*p* = 0.014)		
Left IFC	RCs > Simple Sentences (*p* < 0.01)			Bilingual > Monolingual in OR (*p* = 0.043); OR > SVO (*p* < 0.01) and PAS (*p* = 0.019) in bilingual

#### Comparison Between Monolingual and Bilingual Adults

Separate two-level MLMs were performed for adult participants for each ROI exploring fixed factors including Language Group, Sentence Type, Sentence Form, Sentence Structure, and the interaction of Language Group x Sentence Type. The power of the models for each ROI varied between 75.7 and 99.9%. The comparisons between monolingual and bilingual adults yielded greater power than the comparisons between monolingual and bilingual children because the AUC differences were larger.

**Figure 4:**
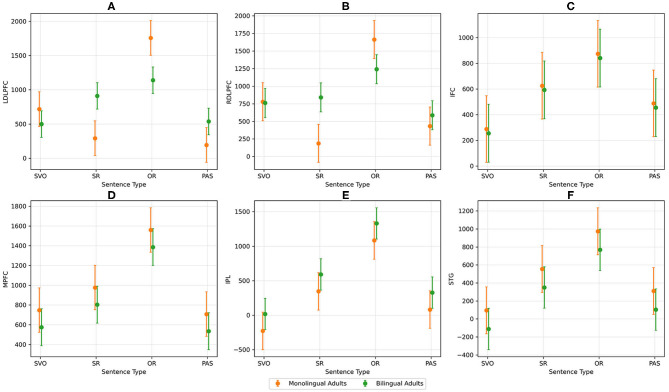
AUC activation for each ROI and sentence type between monolingual and bilingual adults. Picture **(A)** shows the AUC activation for left DLPFC, **(B)** shows the AUC activation for right DLPFC, **(C)** shows the AUC activation for left IFC, **(D)** shows the AUC activation for MPFC, **(E)** shows the AUC activation for left IPL, and **(F)** shows the AUC activation for left STG.

MLM revealed Sentence Structure as a significant predictor across almost all ROIs: left DLPFC [χ^2^(1) = 15.39; *p* < 0.001], right DLPFC [χ^2^(1) = 6.36; *p* = 0.017], MPFC [χ^2^(1) = 18.65; *p* < 0.001], left STG [χ^2^(1) = 7.85; *p* < 0.01], and left IPL [χ^2^(1) = 22.87; *p* < 0.001]. Neuroimaging results showed Sentence Type was a significant predictor for left DLPFC [χ^2^(1) = 6.77; *p* < 0.01], right DLPFC [χ^2^(1) = 7.55; *p* < 0.01], MPFC [χ^2^(1) = 3.93; *p* = 0.047], and left IPL [χ^2^(1) = 26.87; *p* < 0.001]. Additionally, results revealed Sentence Form as a significant predictor for left DLPFC [χ^2^(1) = 4.51; *p* = 0.034] and right DLPFC [χ^2^(1) = 5.95; *p* = 0.015], and Sentence Type x Language Group interaction as a significant predictor for left DLPFC [χ^2^(1) = 4.49; *p* = 0.034] and right DLPFC [χ^2^(1) = 4.56; *p* = 0.033], as delineated in Figure 4.

Thus, greater activation was observed for identifying agents in relative clause sentences as compared to simple sentences across all ROIs except for left IFC. We found greater activation for noncanonical sentences than canonical sentences in the left and right DLPFC. Greater activation was observed for OR sentences than the three other sentence types in left and right DPFC, left IPL and MPFC. For the Sentence Type x Language Group interaction in left and right DLPFC, OR sentences showed greater activation than the other three sentence types for monolinguals. SR showed greater activation in left DLPFC than SVO and PAS for bilinguals (see Table 5).

**Table 5:** Main effects of sentence structure, sentence form, sentence type and sentence type x language group interaction across ROIs for monolingual and bilingual adults.

**ROI**	**Sentence structure**	**Sentence form**	**Sentence type**	**Sentence type x language group**
Left DLPFC	RCs > Simple Sentences (*p* < 0.001)	Noncanonical > Canonical (*p* = 0.034)	OR > SVO (*p* < 0.01), SR (*p* < 0.01), and PAS (*p* < 0.001)	OR > SVO (*p* = 0.013), SR (*p* < 0.01), and PAS (*p* < 0.01) in Monolingual; SR > SVO (*p* = 0.04) and PAS (*p* = 0.028) in Bilingual
Right DLPFC	RCs > Simple Sentences (*p* = 0.017)	Noncanonical > Canonical (*p* = 0.015)	OR > SVO (*p* = 0.01), SR (*p* < 0.01), and PAS (*p* < 0.01)	OR > SVO (*p* = 0.03), SR (p < 0.01), and PAS (*p* = 0.01) in Monolingual
MPFC	RCs > Simple Sentences (*p* < 0.001)		OR > SVO (*p* < 0.001), SR (*p* = 0.03), and PAS (*p* < 0.001)	
Left STG	RCs > Simple Sentences (*p* < 0.01)			
Left IPL	RCs > Simple Sentences (*p* < 0.001)		OR > SVO (*p* < 0.001), SR (p = 0.014), PAS (*p* < 0.01)	

#### Comparison Between Bilingual Adults and Children

Separate two-level MLMs were performed for bilingual children and bilingual adults for each ROI exploring fixed factors included Age Group, Sentence Type, Sentence Form, Sentence Structure, and the interaction of Age Group x Sentence Type. The power of the models for each ROI varied between 75.1 and 98%. The comparisons between bilingual adults and children yielded greater power than the comparisons between monolingual and bilingual children because the AUC differences were larger.

**Figure 5:**
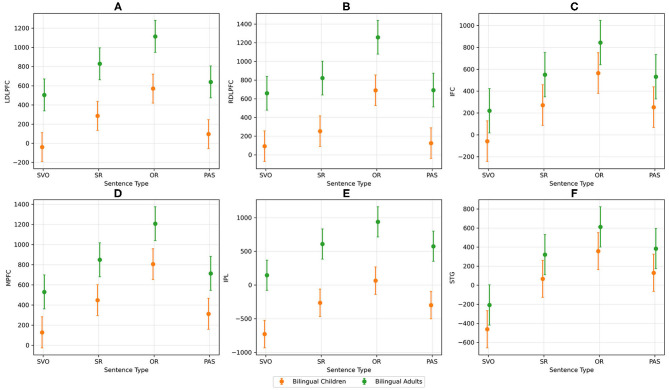
AUC activation for each ROI and sentence type between bilingual children and adults. **(A)** shows the AUC activation for left DLPFC, **(B)** shows the AUC activation for right DLPFC, **(C)** shows the AUC activation for left IFC, **(D)** shows the AUC activation for MPFC, **(E)** shows the AUC activation for left IPL, and **(F)** shows the AUC activation for left STG.

Sentence Structure was found as a significant predictor across all ROIs: left DLPFC [χ^2^(1) = 14.78; *p* < 0.001], right DLPFC [χ^2^(1)= 9.53; *p* < 0.01], MPFC [χ^2^(1) = 13.54; *p* < 0.001], left STG [χ^2^(1) = 6.07; *p* = 0.014], left IPL [χ^2^(1) = 7.95; *p* < 0.01], and left IFC [χ^2^(1) = 6.07; *p* = 0.014]. MLM revealed Sentence Form as a significant predictor for left STG [χ^2^(1) = 4.71; *p* = 0.030], and Age Group as a significant predictor for left DLPFC [χ^2^(1) = 8.94; *p* < 0.01], right DLPFC [χ^2^(1) = 7.22; *p* < 0.01], MPFC [χ^2^(1) = 7.85; *p* < 0.01], and left IPL [χ^2^(1) = 7.67; *p* < 0.01]. Additionally, Sentence Type was a significant predictor for right DLPFC [χ^2^(1) = 12.45; *p* < 0.001] and MPFC [χ^2^(1) = 8.95; *p* < 0.01]. No main effects were found for interactions, as delineated in Figure 5.

In general, greater activation was observed for relative clauses across all ROIs compared with simple sentences. Bilingual adults demonstrated greater activation in left and right DLPFC, MPFC, and left IPL than bilingual children. OR sentences demonstrated greater neural recruitment than the other three sentence types in right DLPFC and MPFC. Greater activation was observed for noncanonical sentences in left STG compared with canonical sentences (see Table 6).

**Table 6:** Main effects of sentence structure, sentence form, and age group across ROIs for bilingual adults and children.

**ROI**	**Sentence structure**	**Sentence type**	**Sentence form**	**Language group**
Left DLPFC	RCs > Simple Sentences (*p* < 0.001)			Adults > Children (*p* < 0.01)
Right DLPFC	RCs > Simple Sentences (*p* < 0.01)	OR > SVO (*p* < 0.001), SR (*p* < 0.01), and PAS (*p* < 0.001)		Adults > Children (*p* < 0.01)
MPFC	RCs > Simple Sentences (*p* < 0.001)	OR > SVO (*p* < 0.001), SR (*p* = 0.03), and PAS (*p* < 0.01)		Adults > Children (*p* < 0.01)
Left STG	RCs > Simple Sentences (*p* = 0.014)		Noncanonical > Canonical (*p* = 0.03)	
Left IPL	RCs > Simple Sentences (*p* < 0.01)			Adults > Children (*p* < 0.01)
Left IFC	RCs > Simple Sentences (*p* = 0.014)			

## Discussion

The purpose of this exploratory study was to investigate whether Chinese-English bilinguals and English monolinguals process simple and complex sentence structures in English differently. We were interested to learn whether AoL2A or the length of L2 exposure predicted the performance of sentence processing in both behavioral assessments and fNIRS measures of the hemodynamic response function. Toward these ends, we examined the performance of Chinese bilinguals (children and adults) and English monolinguals (children and adults) on a syntactic processing task consisting of four sentence types (i.e., SVO, SR, OR, and PAS). As expected, we found a main effect of sentence type for all groups, with identification accuracy for canonical sentences (SVO and SR) being significantly higher than noncanonical sentences (OR and PAS). No significant differences were found between SVO and SR, or between OR and PAS sentences. Interestingly, although both monolingual and simultaneous bilingual children performed significantly better than sequential bilingual children on a test of grammatical judgement during language tests, no significant differences were found between monolingual and sequential bilinguals or between simultaneous bilingual and sequential bilingual children on the agent-selection task.

Research indicates that individuals tend to use both semantic and syntactic plausibility knowledge to facilitate sentence comprehension (Ferreira, 2003; Traxler and Tooley, 2007). For example, to examine effects of agent vs. patient plausibility on sentence comprehension, Ferreira (2003) asked university students to identify the thematic roles of agent in canonical (active) and noncanonical (passive) sentences. Results showed that both sentence type (active vs. passive) and the plausibility of agent/patient relationships influenced the accuracy of agent identification. Therefore, using semantically implausible sentences adopted from Montgomery et al. (2016) avoided semantic cues requiring the participants to make decisions based on syntactic knowledge or linguistic cues. Our exploratory findings show that although sequential bilingual children had significantly different levels of English grammatical knowledge, they demonstrated similar performance on the syntactical processing task. This similar performance on the online task may reveal comparable levels of syntactic knowledge in monolingual and simultaneous bilingual children in the 9–12 years range. However, another possible reason for the contradictory findings between the performance of grammatical knowledge and syntactic processing might be the significant difference between monolinguals and bilinguals' maternal education. Because maternal education for bilinguals was significantly higher than that for monolinguals, these bilinguals may have developed syntactic awareness similarly to the monolinguals. While similar behavioral performances among groups suggest similar neural cognitive operations, the fNIRS analysis compared neural activation patterns in various cortical areas among groups to determine if and how syntactic processing might vary among participants.

### Neuroimaging Results for the Agent-Selection Task

In addition to significant behavioral differences between noncanonical (OR and PAS) and canonical (SVO and SR) sentence types, neuroimaging results revealed significant differences in the hemodynamic response functions obtained as participants processed relative clause sentences (SR and OR) and simple sentences (SVO and PAS). Most previous studies have focused on canonical/noncanonical comparisons, either between SVO and PAS (Ferreira, 2003; Yokoyama et al., 2006) or between SR and OR (Traxler and Tooley, 2007; Kovelman et al., 2008; Jasińska and Petitto, 2013). Less is known about the effect of relative clauses on sentence comprehension compared with simple sentences among monolinguals or bilinguals, especially for bilinguals whose L1 (Chinese) is structured differently from their L2 (English). Because relative clauses in Chinese tend to be shorter, less complex, and follow the head-final model as compared with those in English (Lin, 2011), we expected that bilingual groups would evidence more neural recruitment of areas related to cognitive executive function (i.e., DLPFC) for SR and OR sentence types.

As predicted, in right DLPFC and left IFC, bilingual children evidenced larger AUC HbO values associated with more neural activation for OR sentences as compared with SVO and PAS sentences. Additionally, the hemodynamic response function to OR sentences among bilingual children was greater than that of monolingual children. In left DLPFC, bilingual adults evidenced larger HbO hemodynamic response functions for SR sentences than for SVO and PAS sentences. The findings support the hypothesis that Chinese bilinguals may evidence greater neural recruitment of executive functions for relative clauses because of their L1's sentence structure. Moreover, the differences between bilingual adults (greater activation for SR) and children (greater activation for OR) suggest the impact of longer vs. shorter L1 exposure on RC processing in L2. In Chinese, rather than OR, SR follows the marked noncanonical verb-patient-agent order. Therefore, it is reasonable to expect that SR sentences would be more difficult for Chinese-English bilinguals to process. Because L2 processing of bilinguals may be influenced by their L1 (Abutalebi, 2008; Waldron and Hernandez, 2013), bilingual adults who have longer L1 exposure than children were more likely to recruit greater oxygenated hemoglobin to process SRs in English because of varying sentence structure in Chinese. This finding aligns with the L1 transfer hypothesis. Meanwhile, because RCs in English are longer and more complex than in Chinese, it is reasonable to assume that bilinguals would increase recruitment of neural resources related to executive function when processing the same noncanonical OR sentences. Our finding that bilingual children evidence a greater hemodynamic response in right DLPFC and left IFC for ORs than monolingual children aligns with the hypothesis that L1 influences L2 sentence processing.

### The Effect of AoL2A on Neural Activation Patterns

Prior research has shown that compared to late exposure to another language, early exposure to L2 may lead to increased language competence, such as a native-like accent and greater L2 word-retrieval (Wattendorf et al., 2014). Studies assessing neural activation patterns of bilinguals during language tasks have shown that early/simultaneous bilinguals tend to perform similarly to monolinguals, and late/sequential bilinguals evidence more neural activity related to cognitive control (Jasińska and Petitto, 2013; Berken et al., 2015; Kousaie et al., 2017). Thus, we expected that sequential bilinguals would have a greater hemodynamic response in areas related to cognitive control compared to simultaneous bilinguals. However, one of the most surprising findings of this study was the increased neural recruitment of right DLPFC and left IPL for simultaneous bilinguals compared with sequential bilinguals. More activation of right DLPFC is thought to indicate more cognitive control for simultaneous bilinguals, which seems contrary to prior studies of simultaneous bilinguals. However, we argue that greater activation of neural regions (i.e., DLPFC) related to cognitive control in this study suggests higher L2 competence among the simultaneous bilinguals. Recall that the current study adopted sentence stimuli that are semantically implausible. Research for monolinguals has shown that sentence implausibility requires participants to devote extra cognitive effort (Ferreira, 2003; Traxler and Tooley, 2007). DLPFC supports basic cognitive selection and response (Corbetta and Shulman, 2002), and in the present study, there was greater recruitment of oxygenated hemoglobin in right DLPFC, although not statistically significant, for monolinguals than for sequential bilinguals. Research has shown that bilinguals with high language competence tend to be as sensitive as monolinguals to sentence implausibility (Dussias and Piñar, 2010). In their study to examine the effect of cognitive characteristics on accessing plausibility information among bilingual and monolingual university students, Dussias and Piñar (2010) found that only L2 participants with higher language competence behaved similarly to the monolingual group with longer processing time observed for implausible situations. Thus, more activation of DLPFC for simultaneous bilinguals was expected.

Additionally, similar activation patterns between simultaneous bilinguals and monolinguals in left IPL in the present study reflect greater L2 competence of simultaneous bilinguals as compared with sequential bilinguals. Studies targeting the involvement of IPL in L2 learning have shown that increased left IPL activation was associated with higher L2 proficiency (Mechelli et al., 2004; Grogan et al., 2009; Della Rosa et al., 2013). Therefore, although behavioral data in the present study did not reveal group differences in agent-selection accuracy, the neuroimaging data showed group differences in sentence processing. Greater recruitment of oxygenated hemoglobin in right DLPFC and left IPL for simultaneous bilinguals supports the hypothesis that early exposure to L2 corresponds with neural changes that facilitate L2 processing. A recommendation for future studies could be to examine the effect of AoL2A on sentence processing among bilingual participants with different L1s on the same syntactic task.

### Neural Activation and the Length of L2 Experience

The different hemodynamic response patterns between bilingual adults and children could relate to developmental neural changes associated with age (Jasińska and Petitto, 2013). Prior research with monolinguals has found greater activation of left IFG and left IPL in adults than in children on a lexical-semantic decision task (Moore-Parks et al., 2010). Moore-Parks et al. (2010) argued that the greater neural recruitment for bilingual adults related to increased “top-down” control. Similarly, greater activation of left IPL for bilingual adults was observed in this study, which corresponded with higher L2 proficiency of adults compared with children. Therefore, it was reasonable to expect greater hemodynamic response functions of DLPFC and MPFC for bilingual adults in this study because bilingual adults with higher language proficiency tend to be more sensitive to the plausibility of sentences. As predicted, bilingual adults revealed greater recruitment of oxygenated hemoglobin in right and left DLPFC, and MPFC than children.

### Limitations

Beyond the planned specifications (e.g., semantic implausible sentence stimuli for four sentence types and Chinese-English bilinguals) imposed above, there were other factors that may have influenced the results. The present study focused on brain regions associated with language processing and cognitive control, which are mainly in the frontal area and left hemisphere. However, targeting these “classic language” areas in left hemisphere does not imply the exclusion of the right hemisphere in language processing. Prior studies have shown that brain regions in the right hemisphere, such as right STG and IPL were also involved in language processing (DevousSr et al., 2006; Jasińska and Petitto, 2013, 2014). Examining both the left and right hemisphere may yield more significant differences than found in this study. Another possible limitation of this study is the lack of off-line tests that examine participants' syntactic knowledge more specifically. The grammatical judgement test focused on participants' grammatical knowledge that includes both syntactic and semantic knowledge. In addition to the grammatical judgement test, an off-line behavioral test that could examine participants' syntactic knowledge may reveal different performance across bilingual and monolingual groups. Finally, this exploratory study had some limited statistical power because of the modest sample size and other population parameters including between group correlations and variances. Therefore, readers should be careful about generalizing some of the significant comparative findings to the larger population of Chinese-English bilinguals.

## Conclusion

This exploratory study examined the hemodynamic response patterns among Chinese-English bilingual and English monolingual children and adults as they performed a sentence processing task. We presented the participants with semantically implausible sentence stimuli by including only inanimate nouns. The behavioral results suggest that the groups of children had similar levels of syntactic knowledge, although with different levels of grammatical knowledge. However, the neuroimaging results revealed different hemodynamic response patterns among groups. Simultaneous bilinguals evidenced greater hemodynamic responses in right DLPFC and left IPL compared to sequential bilingual children, which suggested that AoL2A does matter. Moreover, bilingual adults demonstrated greater neural recruitment of oxygenated hemoglobin in both left and right DLPFC, MPFC and left IPL compared to bilingual children, which indicated that longer vs. shorter L2 experience yields neural changes in developing brain. One of the surprising findings was greater recruitment of neural resources for relative clauses in all ROIs, especially for bilingual participants. The results suggested the possibility of the effect of L1 knowledge on L2 sentence processing.

The present study offers new insights into the effect of L1 knowledge on L2 sentence processing that may facilitate researchers' efforts to develop language tasks that adapt to learners' language backgrounds. Additionally, this study used semantic implausible sentences to constrain participants to use syntactic knowledge to make decisions, which is novel in neuroimaging studies with interesting results. We recommend that bilinguals with different L1s be included in future studies to investigate the effect of syntactical knowledge measured by avoiding the influence of semantic cues.

## Data Availability Statement

The raw data supporting the conclusions of this article will be made available by the authors, without undue reservation.

## Ethics Statement

The studies involving human participants were reviewed and approved by Utah State University's Human Research Protection Office. Written informed consent to participate in this study was provided by the participants' legal guardian/next of kin.

## Author Contributions

RG conceived of and designed the study, obtained the funding for the study, and provided the language comprehension tasks from his prior research with James Montgomery and Julia Evans. AH, CO, SJ, RW, and GD recruited the subjects, collected the data, processed the data, and organized the database. GD performed the statistical analysis and wrote the first draft of the manuscript. GD and KM collaborated on drafting the manuscript. KM and RG revised it critically for important intellectual content. All authors contributed to manuscript revision, read, and approved the submitted version.

## Conflict of Interest

The authors declare that the research was conducted in the absence of any commercial or financial relationships that could be construed as a potential conflict of interest.

## Supplementary Material

Supplementary Figure 1 Deoxyhemoglobin curves for monolingual and bilingual adults.

Supplementary Figure 2 Oxyhemoglobin curves for monolingual and bilingual adults.

Supplementary Figure 3 Deoxyhemoglobin curves for bilingual children and adults.

Supplementary Figure 4 Oxyhemoglobin curves for bilingual children and adults.

Supplementary Figure 5 Deoxyhemoglobin curves for monolingual and bilingual children.

Supplementary Figure 6 Oxyhemoglobin curves for monolingual and bilingual children.

Supplementary Figure 7 Hemodynamic curves for left IFC and STG activation for each individual across sentence types.
