# Supplementary figures and images for: Use of Functional Near Infrared Spectroscopy to Assess Syntactic Processing by Monolingual and Bilingual Adults and Children
Source: Front Hum Neurosci. 2021 Feb 3;15:621025. doi: 10.3389/fnhum.2021.621025 (PMC7902003; doi:10.3389/fnhum.2021.621025)

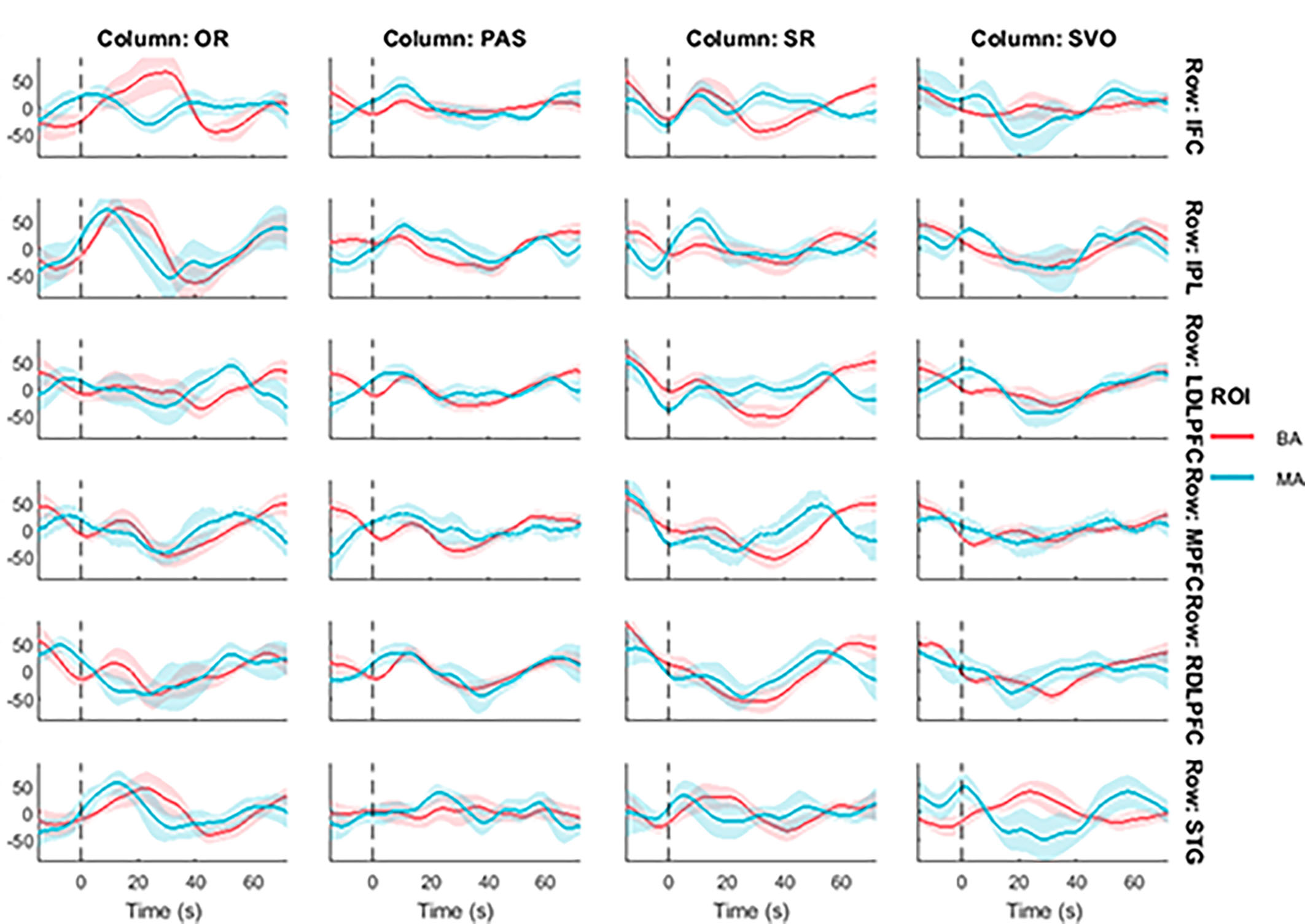

Supplement: Supplementary Figure 1 — Deoxyhemoglobin curves for monolingual and bilingual adults. [file Image_1.jpg]

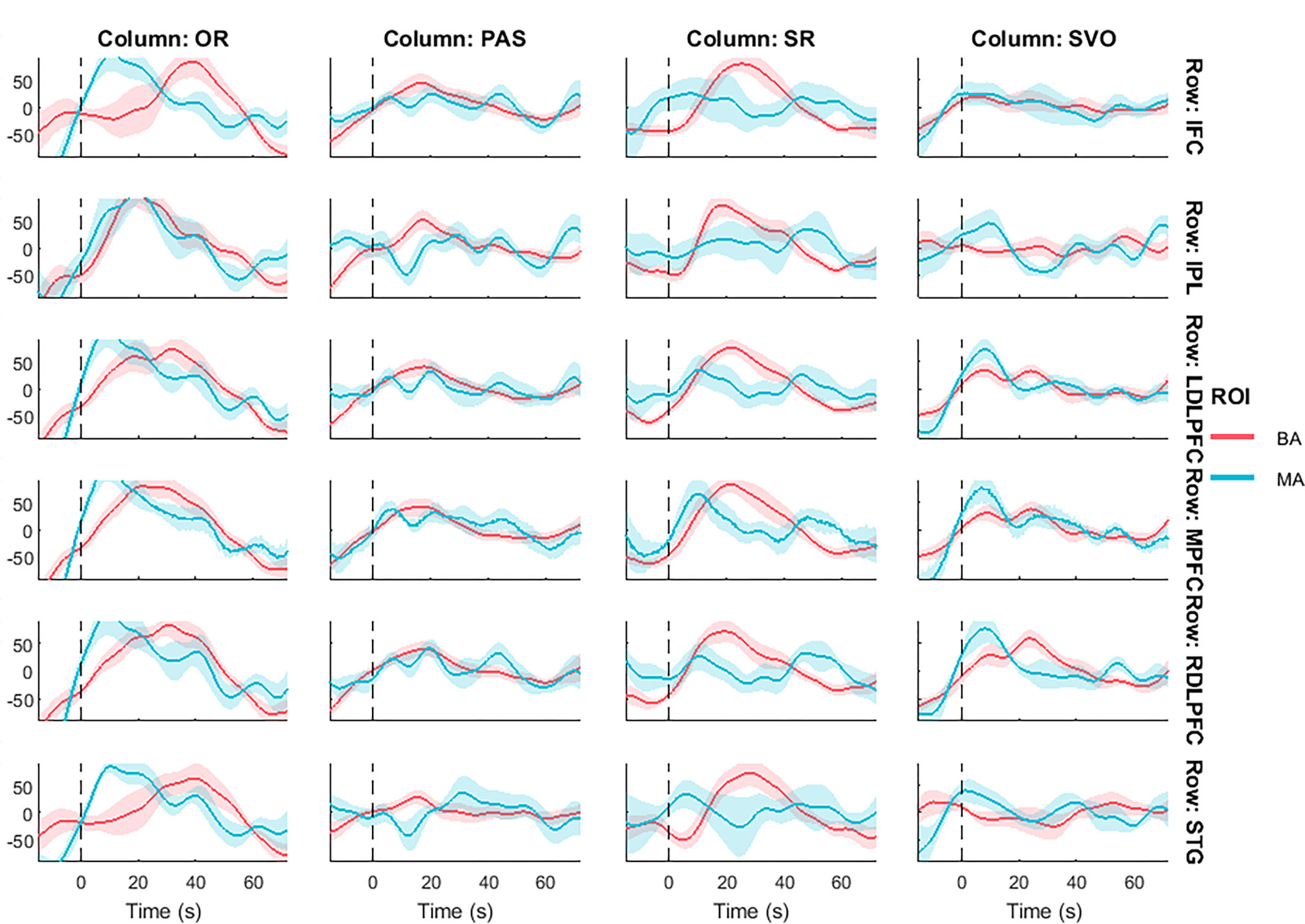

Supplement: Supplementary Figure 2 — Oxyhemoglobin curves for monolingual and bilingual adults. [file Image_2.jpg]

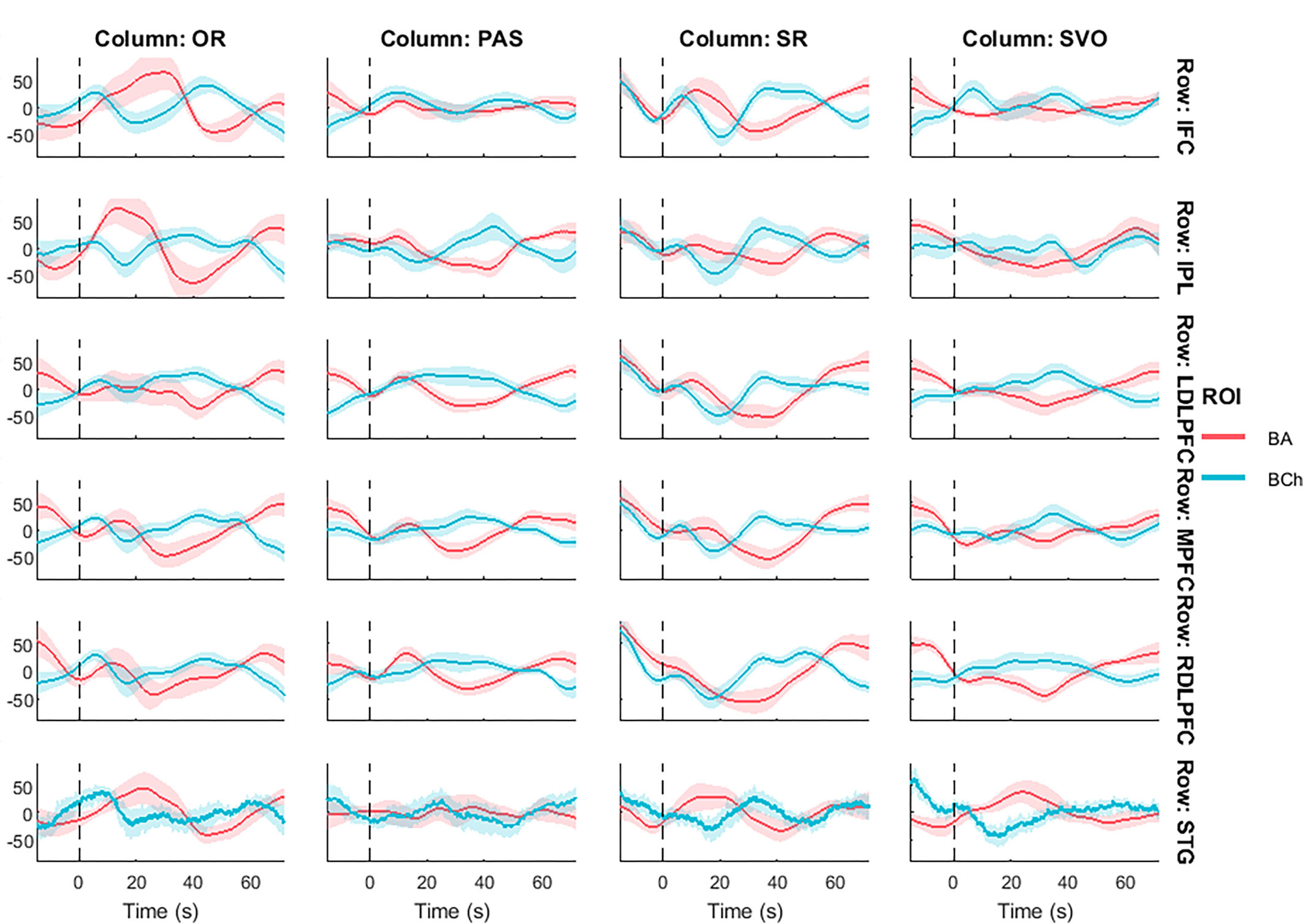

Supplement: Supplementary Figure 3 — Deoxyhemoglobin curves for bilingual children and adults. [file Image_3.jpg]

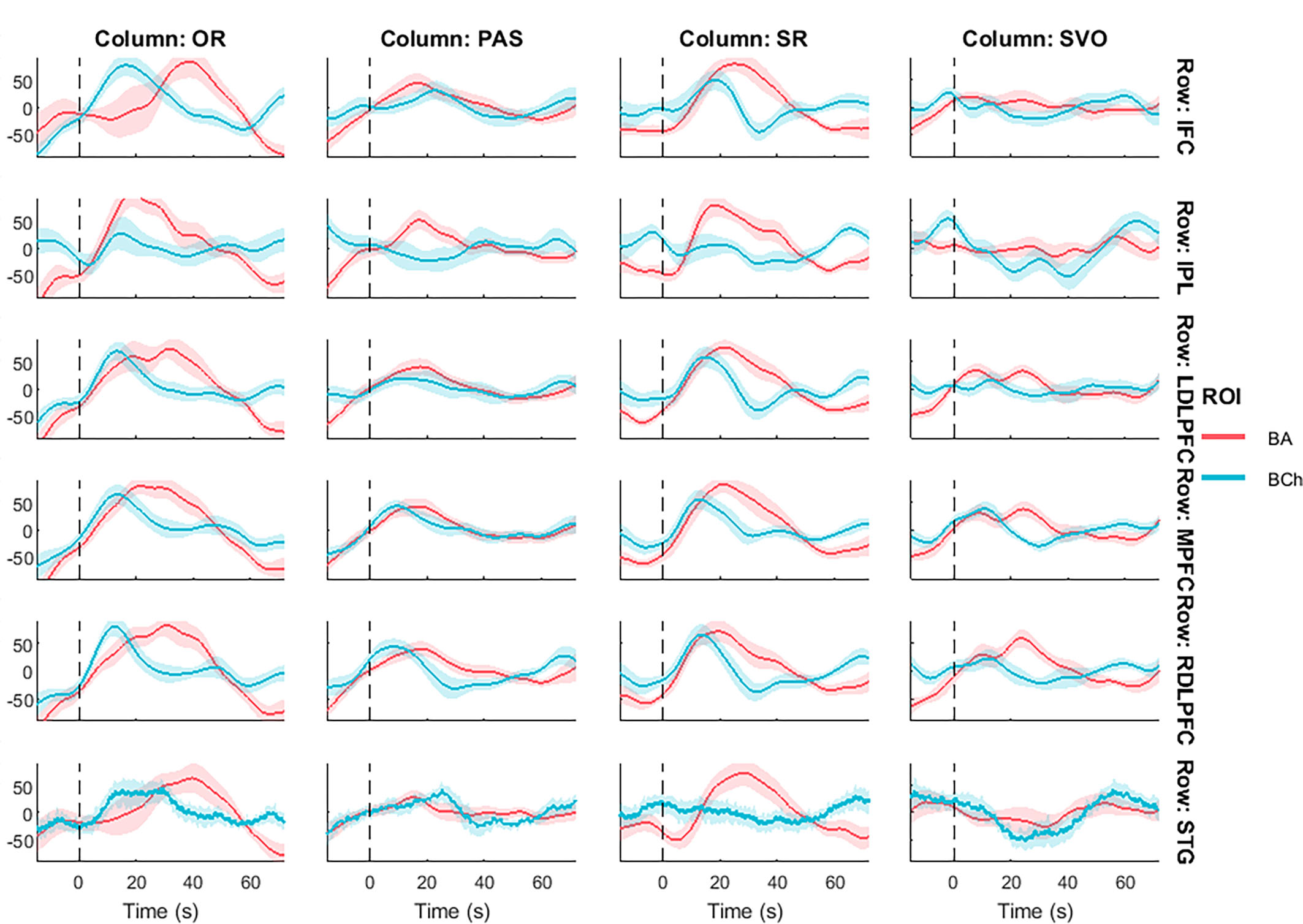

Supplement: Supplementary Figure 4 — Oxyhemoglobin curves for bilingual children and adults. [file Image_4.jpg]

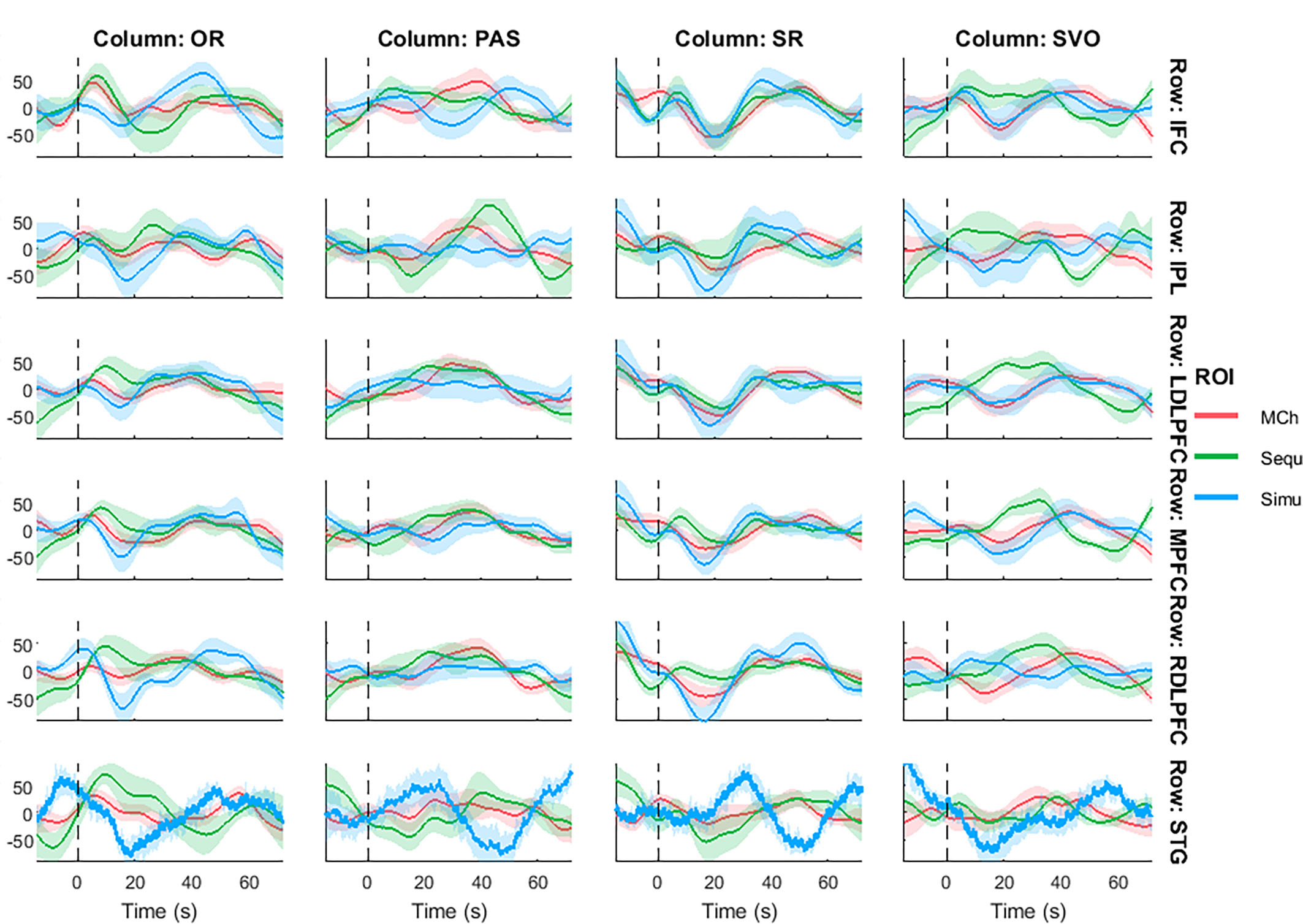

Supplement: Supplementary Figure 5 — Deoxyhemoglobin curves for monolingual and bilingual children. [file Image_5.jpg]

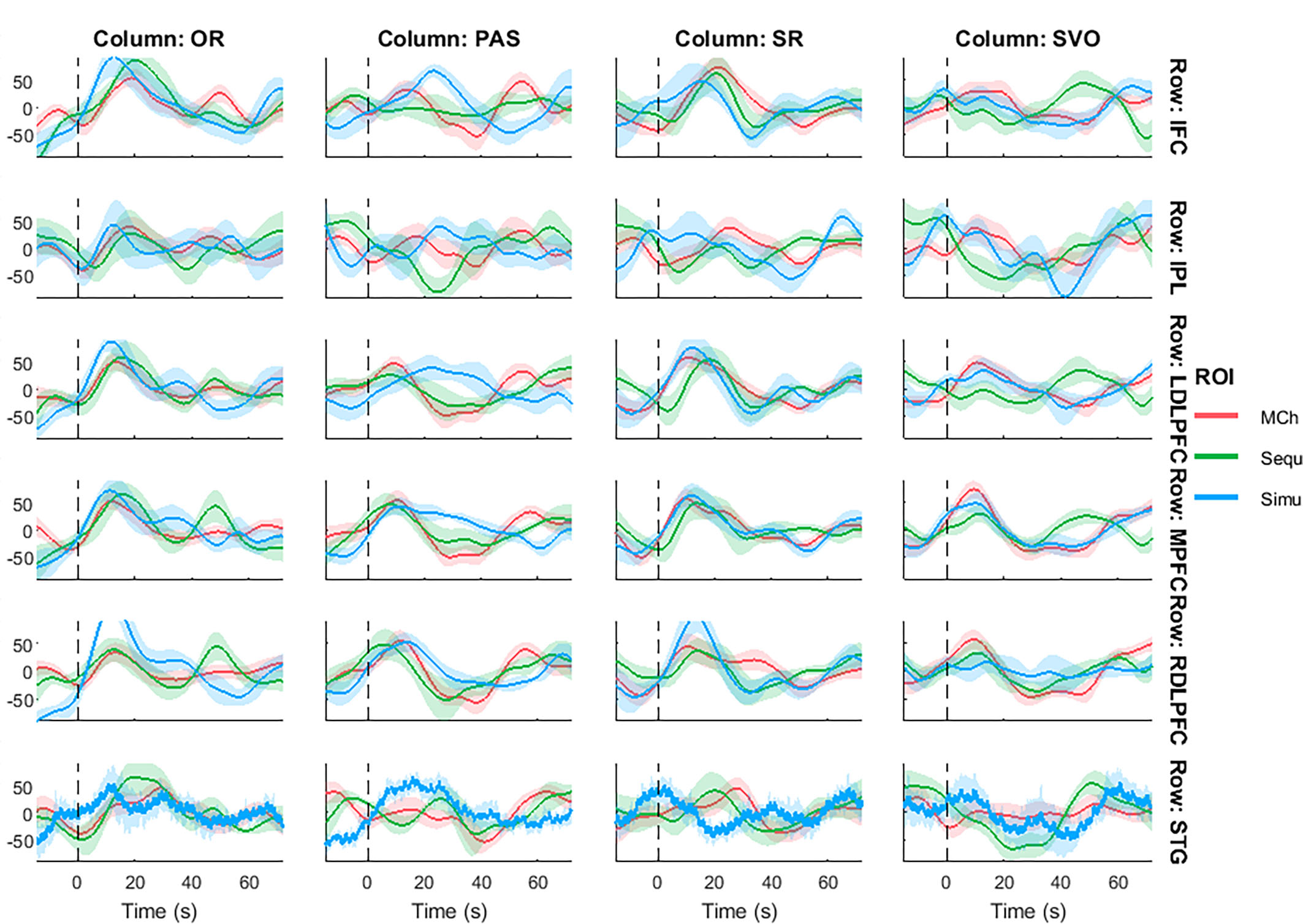

Supplement: Supplementary Figure 6 — Oxyhemoglobin curves for monolingual and bilingual children. [file Image_6.jpg]

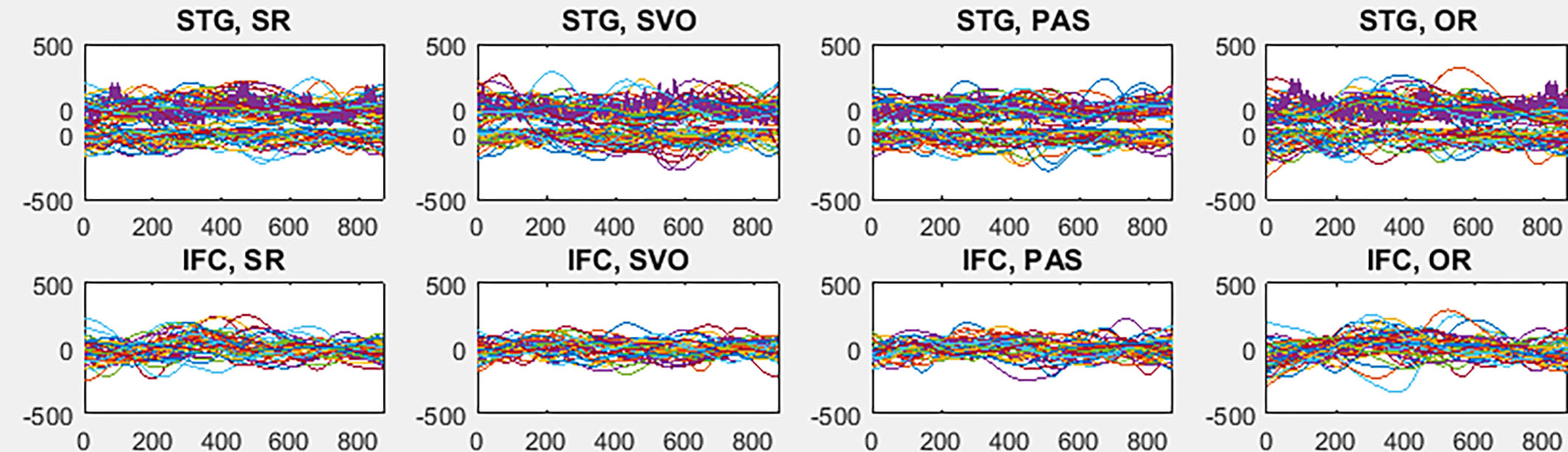

Supplement: Supplementary Figure 7 — Hemodynamic curves for left IFC and STG activation for each individual across sentence types. [file Image_7.jpg]
