# Supplementary material for: Use of Functional Near Infrared Spectroscopy to Assess Syntactic Processing by Monolingual and Bilingual Adults and Children
Source: Front Hum Neurosci. 2021 Feb 3;15:621025. doi: 10.3389/fnhum.2021.621025 (PMC7902003; doi:10.3389/fnhum.2021.621025)
Supplement: Supplementary file 8 [file Table_1.docx]

| **Table 1**  ***Power of Fit Model for Each ROI (Monolingual Children vs. Bilingual Children)*** | | |
| --- | --- | --- |
| ROI | Fit Model | Power |
| Left  DLPFC | AUC ~ Sentence Structure + Group + (1\|Participant) | 64.7% (n = 124) |
| Right DLPFC | AUC ~ Sentence Type + Sentence Structure + Group + Sentence Type * Group + (1\|Participant) | 67.2% (n = 124) |
| MPFC | AUC ~ Sentence Structure + Group + (1\|Participant) | 67.2% (n = 124) |
| Left STG | AUC ~ Sentence Structure + Group + (1\|Participant) | 76.4% (n = 124) |
| Left IPL | AUC ~ Sentence Type + Group + (1\|Participant) | 52.4% (n = 124) |
| Left IFC | AUC ~ Sentence Structure + Group + Sentence Type * Group + (1\|Participant) | 68.9% (n = 124) |
|  |  |  |
| *Note. The power was generated with 95% Confidence Interval and a fixed effect size of -0.05.* | | |
