# Supplementary material for: Use of Functional Near Infrared Spectroscopy to Assess Syntactic Processing by Monolingual and Bilingual Adults and Children
Source: Front Hum Neurosci. 2021 Feb 3;15:621025. doi: 10.3389/fnhum.2021.621025 (PMC7902003; doi:10.3389/fnhum.2021.621025)
Supplement: Supplementary file 10 [file Table_3.docx]

| **Table 3**  ***Power of Fit Model for Each ROI (Bilingual Children vs. Bilingual Adults)*** | | |
| --- | --- | --- |
| ROI | Fit Model | Power |
| Left DLPFC | AUC ~ Sentence Structure + Group + (1\|Participant) | 96.9% (n =112) |
| Right DLPFC | AUC ~ Sentence Type + Sentence Structure + Group + (1\|Participant) | 94.5% (n =112) |
| MPFC | AUC ~ Sentence Type + Sentence Structure + Group + (1\|Participant) | 98% (n =112) |
| Left STG | AUC ~ Sentence Structure + Sentence Form + Group + (1\|Participant) | 91.6% (n =112) |
| Left IPL | AUC ~ Sentence Structure + Group + (1\|Participant) | 76.6% (n = 112) |
| Left IFC | AUC ~ Sentence Structure + Group + (1\|Participant) | 75.1% (n = 112) |
| *Note. The power was generated with 95% Confidence Interval and a fixed effect size of -0.05.* | | |
